# Marine Plastic Pollution in Waters around Australia: Characteristics, Concentrations, and Pathways

**DOI:** 10.1371/journal.pone.0080466

**Published:** 2013-11-27

**Authors:** Julia Reisser, Jeremy Shaw, Chris Wilcox, Britta Denise Hardesty, Maira Proietti, Michele Thums, Charitha Pattiaratchi

**Affiliations:** 1 School of Environmental Systems Engineering, University of Western Australia, Perth, Western Australia, Australia; 2 Oceans Institute, University of Western Australia, Perth, Western Australia, Australia; 3 Wealth from Oceans Flagship, Commonwealth Scientific and Industrial Research Organisation, Floreat, Western Australia, Australia; 4 Centre for Microscopy, Characterisation and Analysis, University of Western Australia, Perth, Western Australia, Australia; 5 Marine and Atmospheric Research, Commonwealth Scientific and Industrial Research Organisation, Hobart, Tasmania, Australia; 6 Instituto de Oceanografia, Universidade Federal do Rio Grande, Rio Grande, Rio Grande do Sul, Brazil; 7 Australian Institute of Marine Science, Perth, Western Australia, Australia; University of Wales Swansea, United Kingdom

## Abstract

Plastics represent the vast majority of human-made debris present in the oceans. However, their characteristics, accumulation zones, and transport pathways remain poorly assessed. We characterised and estimated the concentration of marine plastics in waters around Australia using surface net tows, and inferred their potential pathways using particle-tracking models and real drifter trajectories. The 839 marine plastics recorded were predominantly small fragments (“microplastics”, median length = 2.8 mm, mean length = 4.9 mm) resulting from the breakdown of larger objects made of polyethylene and polypropylene (e.g. packaging and fishing items). Mean sea surface plastic concentration was 4256.4 pieces km^−2^, and after incorporating the effect of vertical wind mixing, this value increased to 8966.3 pieces km^−2^. These plastics appear to be associated with a wide range of ocean currents that connect the sampled sites to their international and domestic sources, including populated areas of Australia's east coast. This study shows that plastic contamination levels in surface waters of Australia are similar to those in the Caribbean Sea and Gulf of Maine, but considerably lower than those found in the subtropical gyres and Mediterranean Sea. Microplastics such as the ones described here have the potential to affect organisms ranging from megafauna to small fish and zooplankton.

## Introduction

Plastics are a diverse group of materials derived from petrochemicals [1]. Their global production has grown exponentially from 1,700,000 tonnes in 1950 to 280,000,000 tonnes in 2011 [2]. The disposability of plastics, together with their low recycling rates, has contributed to a significant rise in the amount of waste produced globally [3]. For instance, in Australia, 1,433,046 tonnes of plastics were used in 2010–2011, of which only 20% was recycled. Moreover, around 37% of this plastic was for the manufacturing of single-use disposable packaging [4]. Plastics are transported from populated areas to the marine environment by rivers, wind, tides, rainwater, storm drains, sewage disposal, and even flood events. It can also reach the sea from vessels (e.g. fishing gear) and offshore installations [5]. Once in the oceans, they will either float at the ocean surface, or sink to the seafloor if made from polymers denser than seawater [6]. Buoyant plastics may be cast ashore by inshore currents or winds [7], or may enter the open ocean, where they tend to accumulate in convergence zones such as the ones formed by the five large-scale gyres (South and North Pacific, South and North Atlantic, and Indian [8]–[10]).

Marine plastics are known to undergo fragmentation into increasingly smaller pieces by photochemical, mechanical and biological processes [6], [11]. Plastics are also directly manufactured in small sizes (<5mm), which may find their way into the oceans. These include virgin plastic pellets (pelletwatch.org; [12]), synthetic fibers from clothes [13], micro beads from cosmetics [14], and synthetic ‘sandblasting’ media [6]. There is increasing awareness that these small plastic particles (often called microplastics when smaller than 5 mm [6]) represent a significant proportion of the human-made debris present in the oceans. However, their at-sea spatial and temporal dynamics remain poorly assessed, mostly due to a lack of data on their characteristics and at-sea occurrence [15], [16]. In Australia, the only published information on microplastics comes from a global study that recorded their occurrence in the sediments of Busselton beach (Western Australia) and Port Douglas (Queensland) [13]. Apart from this, our current knowledge on plastic contamination in the Australian marine environment is restricted to (1) beach litter cleanups that record mainly the occurrence of relatively large objects (e.g. [17]–[19]), (2) land-based surveys of marine megafauna impacted by marine debris (e.g. [17], [20]–[22]), and (3) inferences based on plastic pollution reports from New Zealand (e.g. [23]).

The impacts of plastics on marine vertebrates, such as turtles, mammals and birds, have been well recognized since the 80's [24], [25]. However, only recently has concern about the effects of small plastic particles on food webs and marine ecosystems been raised. More than half of modern plastics contain at least one hazardous ingredient [26] and those that end up in aquatic systems can become increasingly toxic by adsorbing persistent organic pollutants on their surface [27]. These concentrated toxins might then be delivered to animals via plastic ingestion and/or endocytosis [28], [29] and transferred up their food webs [30]–[32]. This bio-magnification process is more likely to happen when plastics are small enough to be ingested by organisms that are close to the bottom of the ocean food web, such as planktivorous fish [33] and zooplankton [34]. For instance, it was inferred that small plastic particles found in the stomach contents of Southern Bluefin tuna captured close to Tasmania [35] were coming from the guts of their prey: myctophid fish [36]. In this scenario, plastic contaminants can be transferred to the affected organism and then biomagnified up the food chain. If this process is taking place, plastics can affect the health of food webs, which include humans as an apex predator.

Australia's acknowledgement of plastic threats to marine ecosystems is mostly limited to impacts from relatively large debris (e.g. abandoned fishing nets, plastic bags) on marine megafauna (e.g. turtles, mammals, birds) [37]. A first step towards a better understanding of the extent of marine plastic hazards to Australian organisms and environments is a better assessment of the occurrence and characteristics of plastic debris at-sea. To this end, we characterized (size, type, color, polymer) and estimated concentration (pieces km^−2^) of plastics in waters around Australia using surface net tows. Additionally, potential pathways taken by the collected plastics were inferred using outputs of a dispersal model and trajectories of satellite-tracked drifting buoys.

## Materials and Methods

### Ethics Statement

Permits to conduct this field research were obtained from the Great Barrier Reef Marine Park Authority (GBRMPA: permit G11/34378.1). No other special permitting was required because sampling was limited to the collection of marine debris.

**Figure 1:**
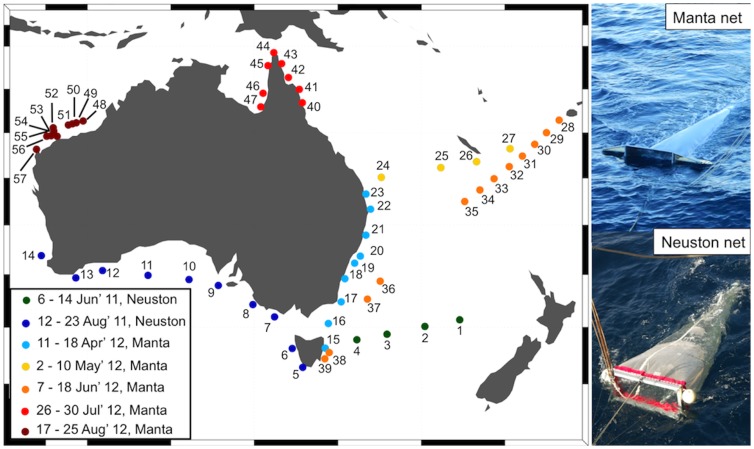
Location of the 57 net stations sampled during this study. Dot colors indicate the voyage when the net station was sampled and numbers follow the chronological order of sampling. Pictures of the two types of net used are shown in the right panel.

During seven transit voyages aboard Australian vessels (Figure 1), we undertook three consecutive 15-minute net tows (mean ± standard deviation tow length = 1.3±0.50 km) at 57 locations (hereafter called “net stations”), while the ship was travelling at a speed of 2 – 4 knots. These net tows sampled the air-sea interface, using a Neuston net (1.2×0.6 m mouth, 335 µm mesh) or a Manta net (1×0.17 m mouth, 333 µm mesh). After each net tow, the collected material was transferred to a container filled with seawater and examined for floating plastic pieces for at least an hour by a trained observer (J.R.). Each plastic piece was picked up with forceps and placed in a graduated dish to be counted, measured (length), photographed and classified into type (hard, soft, line, expanded polystyrene, pellet), and color. A random sample of 200 plastic pieces was selected for polymer composition analysis by Fourier transform infrared spectrometry (FT-IR; range = 500 – 4000 cm^−1^). Polymer type was determined by comparing sample FT-IR spectra against known spectra from a database (Perkin-Elmer ATR of Polymers Library).

To estimate sea surface plastic concentrations (*Cs*, pieces km^−2^), we first divided the number of plastic pieces found in the cod-end of each net tow by its towed area, which was estimated by multiplying net mouth width by tow length (determined from GPS position data). Mean *Cs* was then estimated for each of the 57 net stations by averaging the *Cs* of its three net tows. To our knowledge, this is the first study to take net tow replicates for marine plastic sampling. Apart from providing us measurements of *Cs* variability, our approach (i.e. execution of 3 short net tows instead of 1 long trawl) also avoided net clogging by gelatinous zooplankton.

Since buoyant plastics are vertically distributed due to wind-driven mixing, we also estimated depth-integrated plastic concentrations (*Ci*, pieces km^−2^) by applying a one-dimensional column model [15]:
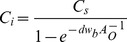



Where:

*d* = immersion depth of the surface-towed net; equal to 0.17 m for the Manta net tows (full immersion of the net frame) and 0.3 m for the Neuston net tows (half of the frame immersed).

*w_b_* = buoyant rise velocity of marine plastics; equal to 0.02 m s^−1^. Preliminary experiments indicate that it ranges from 0.005 – 0.035 m s^−1^ [15].

*A_o_* = near-surface turbulent (eddy) exchange coefficient, which was estimated by:
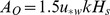



Where:

*k* = von Karman constant; equal to 0.4.

*H_s_* = significant wave height (m).

u_*w_ = frictional velocity of water (m s^−1^).

Both *H_s_* and u_*w_ were taken from the ERA-Interim model [38]. There was a considerable similarity between wind fields of the ERA-Interim forecast model (*U_10_*) and the wind speed measured by an anemometer (*w*) on five of our seven voyages (*U_10_* = 0.85+1.04*w*, *r^2^* = 0.79, N = 39 net stations), indicating that the use of the model outputs is adequate.

To infer potential pathways taken by the collected plastics, we used two approaches: (1) application of the Australian Connectivity Interface Connie2 (csiro.au/connie2), and (2) trajectories of satellite-tracked buoys from the Global Drifter Program (aoml.noaa.gov/phod/dac). In our first approach, an area of 0.1° latitude by 0.1° longitude was created around each net station and particle-tracking models were run backwards in time. Particles were released within these areas over a 30-day period (25 particles per day), and subsequently tracked for a dispersal time equal to 45 days. These models were forced by averaged ocean current fields (2002 – 2006) of the month when the net station was sampled. Details of the particle tracking model, and the eddy-resolving/data-assimilating ocean general circulation model can be found in [39] and [40], respectively. In our second approach, an area of 4° latitude by 4° longitude was centered on each net station and drifters (drogued and un-drogued) that reached these regions were selected. The tracks starting from the drifter release point until they entered one of the net station areas were then plotted onto maps.

## Results

**Figure 2:**
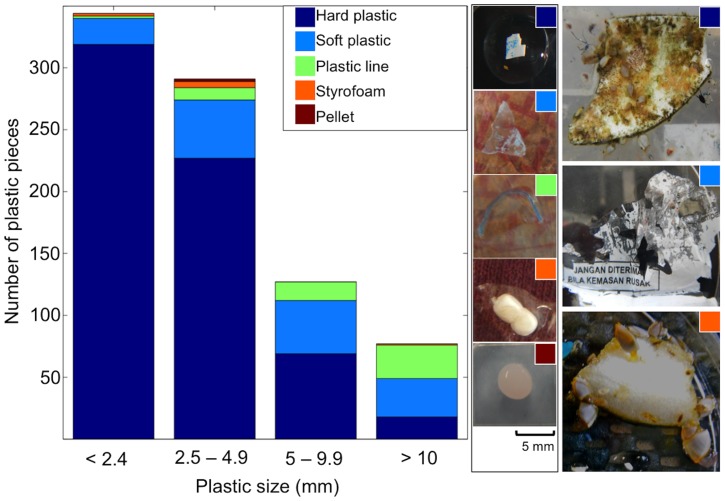
Size and types of marine plastics collected around Australia. Bars indicate the number of plastic pieces within each size category (<2.5, 2.5 – 4.9, 5 – 10, >10 mm) and colors show the amount of each plastic type within size categories. Examples of the types of plastic we collected are shown in the photos, including our biggest fragment of hard plastic (length = 57 mm, net station 32), soft plastic (length = 73 mm, net station 57, note the Indonesian words), and expanded polystyrene (Styrofoam cup fragment, length = 24.3 mm, net station 28).

**Figure 3:**
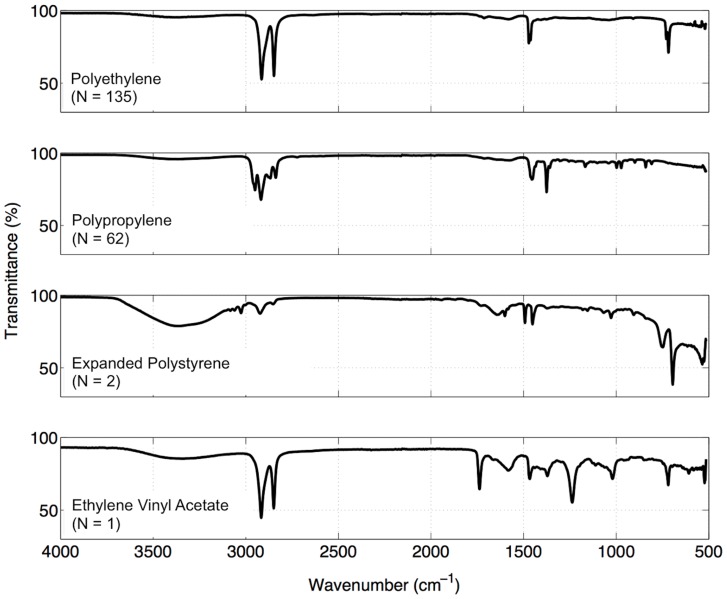
Mean infrared spectra of the plastic pieces within each polymer type.

We recorded 839 pieces of plastic, ranging in length from 0.4 to 82.6 mm (median = 2.8 mm, mean ± standard error = 4.9±0.27 mm, Figure 2). The majority of these plastic pieces had low circularity in their shape when compared to manufactured plastic particles (e.g. pellets and microbeads from cosmetics), suggesting they mostly resulted from the breakdown of larger items. The main plastic type was hard plastic (N = 633, median length = 2.4 mm, range = 0.7 – 57.0 mm) followed by soft plastic (N = 142, median length = 5.0 mm, range = 0.5 – 73.0 mm), plastic line (N = 54, median length = 10.3 mm, range = 2.0 – 82.6 mm), expanded polystyrene (N = 8, median length = 2.9 mm, range = 1.3 – 24.3 mm), and pellet (N = 2, both 4 mm). Most plastics were white/transparent (84.7%), but blue (8.3%) and other colors (7%) were also present. Of the 200 pieces subjected to FT-IR, 67.5% were made of polyethylene, 31% of polypropylene, 1% of expanded polystyrene, and 0.5% of ethylene vinyl acetate (Figure 3).

**Figure 4:**
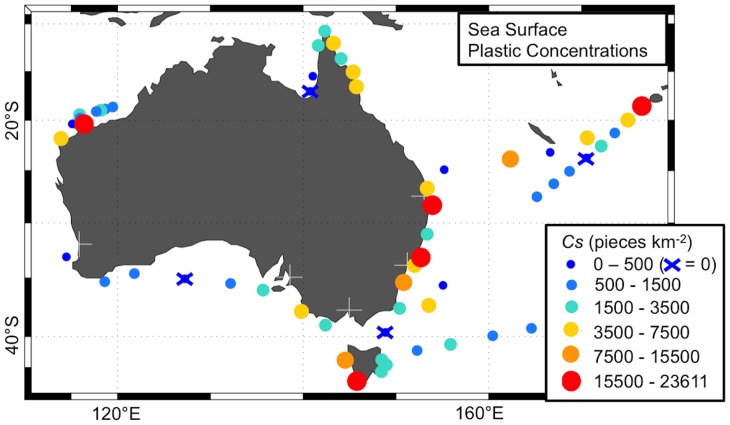
Mean sea surface plastic concentration (*Cs*) at the 57 net stations. White crosses indicate location of major Australian cities (population >1 million). From west to east: Perth, Adelaide, Melbourne, Sydney, and Brisbane.

Approximately 80% of our net tows (136 out of 171), and 93% of our net stations (53 out of 57), had at least one piece of plastic (range: 0 – 68, median = 2, mean ± standard error = 4.9±0.63 pieces per net tow). Estimated sea surface plastic concentrations (*Cs*) for each net tow ranged from 0 to 48895.6 pieces km^−2^ (median = 1932.1 pieces km^−2^, mean ± standard error = 4256.4±757.79 pieces km^−2^) and the mean *Cs* of net stations varied between 0 and 23610.7 pieces km^−2^ (Figure 4, Table S1).

**Figure 5:**
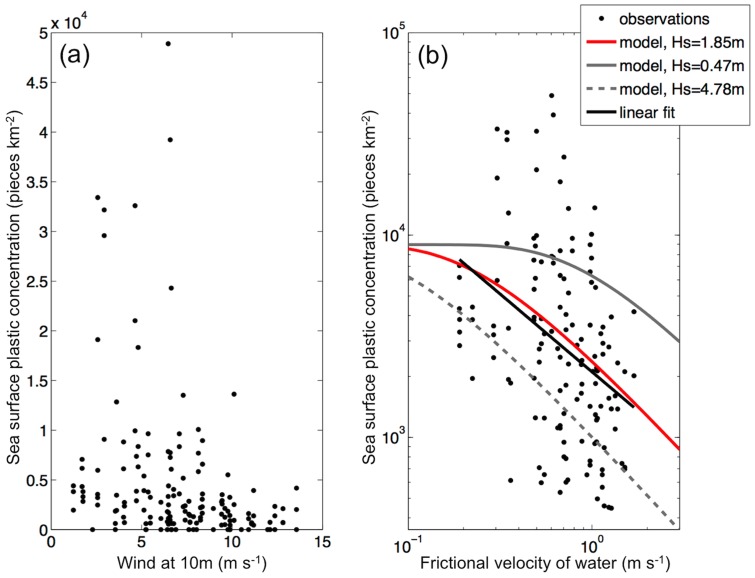
Sea surface plastic concentration (*Cs*) versus a) wind speed (*U_10_*) and b) water friction velocity (u_*w_). In (b) we also show the linear fit (*Cs* = *a* (u_*w_)*^b^*) and theoretical model estimates for *Cs*, when depth-integrated plastic concentration (*C_i_*) is equal to 8966 (mean *Ci* of the 171 net tows) and significant wave height (*Hs*) is equal to the mean (1.85 m), maximum (4.78 m) and minimum (0.47 m) values estimated for the 57 net stations.

**Figure 6:**
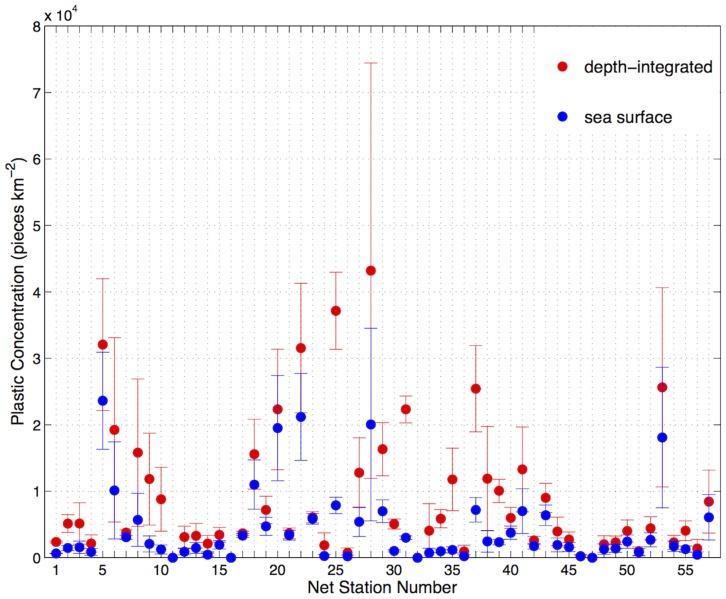
Mean and standard error of sea surface (*Cs)* and depth-integrated (*Ci)* plastic concentrations. Blue represents mean and standard error of *Cs* and red represents mean and standard error of *Ci*.

Relatively high mean *Cs* (>15500 pieces km^−2^) were estimated only at low wind speeds (<7 m s^−1^, Figure 5a). There was an inverse relationship between *Cs* and wind forcing (*b* = −0.77 in *Cs* = *a*(u_*w_)*^b^*), which was relatively consistent with the biophysical model applied here (Figure 5b). When taking into account the effect of wind-mixing, net tow plastic concentrations increased by a mean factor of 2.8 (range: 1.04 – 10.0, median = 1.9). Hence, the amount of plastics collected by our net tows (*Cs*) represents anywhere between 10.0% and 96.1% (median = 52.7%, mean ± standard deviation = 50.0±24.47%) of the estimated total amount of plastic present in the water column (*Ci,* Figure 6).

**Figure 7:**
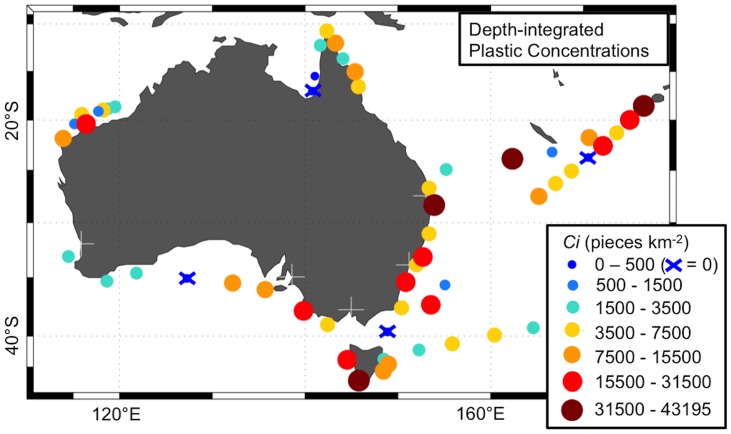
Mean depth-integrated plastic concentration (*Ci*) at the 57 net stations. White crosses indicate location of major Australian cities (population >1 million). From west to east: Perth, Adelaide, Melbourne, Sydney, and Brisbane.

Depth-integrated plastic concentration estimates (*Ci*) for each net tow ranged from 0 to 105438.6 pieces km^−2^ (median = 4363.7 pieces km^−2^, mean ± standard error = 8966.3±1330.75 pieces km^−2^) and the mean *Ci* of net stations ranged from 0 to 43194.5 pieces km^−2^ (Figure 7). In this scenario, plastic concentrations higher than 15500 pieces km^−2^ (red dots) were quite common, and those higher than 31500 pieces km^−2^ (dark red dots) were found close to populated areas (Brisbane and Fiji) as well as in some remote coastal regions (southwest Tasmania) and oceanic areas (Figure 7).

**Figure 8:**
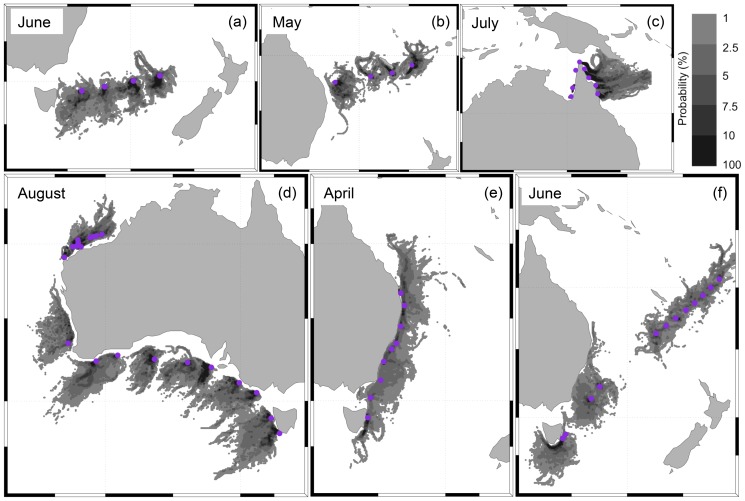
Cumulative probability distribution of virtual particles arriving at the 57 net stations. The month when the virtual particles (25 per day) were released is indicated in each panel. Backtracking dispersal time was equal to 45 days and arriving destinations (net stations) are marked with purple dots. See also Maps S1.

**Figure 9:**
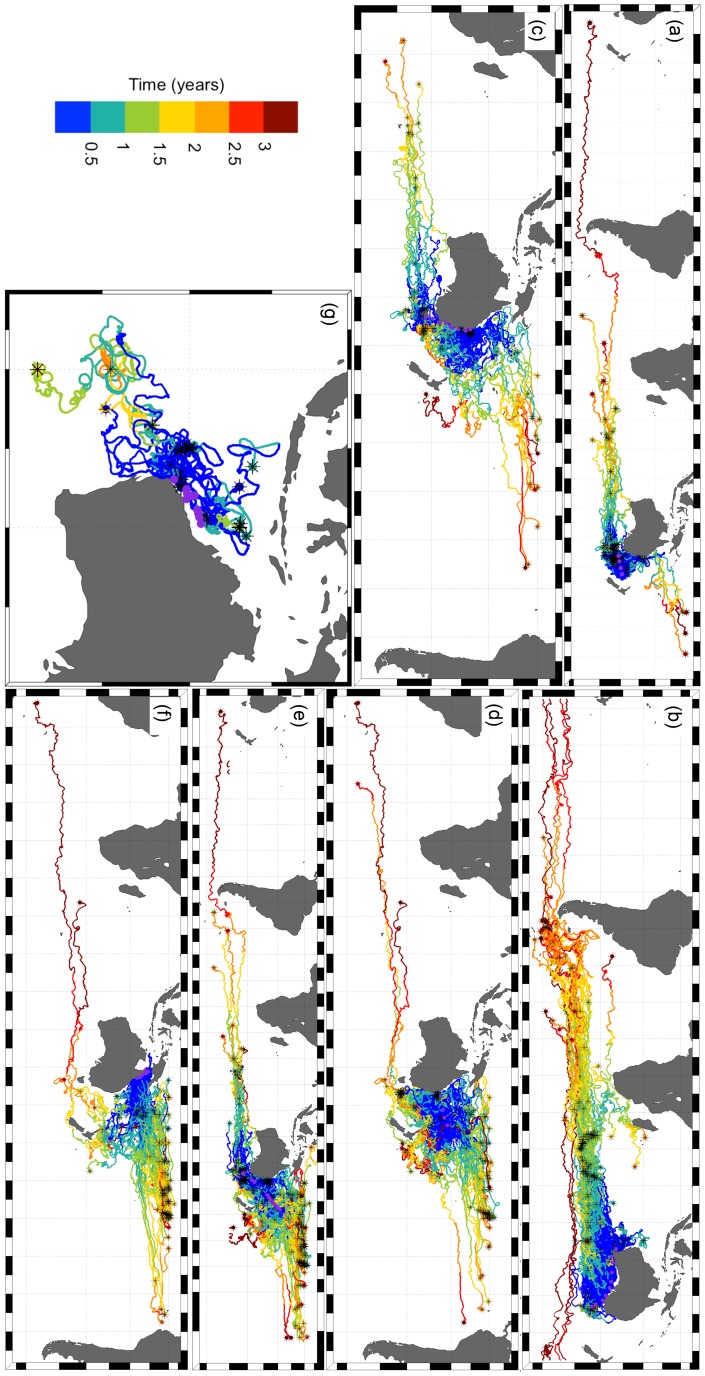
Real drifter pathways arriving at the 57 net stations. Purple dots indicate net station locations and asterisks indicate drifter release areas. See also Maps S2.

A wide range of pathways was taken by the virtual particles arriving at the net stations (Figure 8 and Maps S1). The routes taken by real drifters, from their release points to the net stations, showed similar patterns but covered larger areas due to their longer drifting time and wider range of release date (Figure 9 and Maps S2).

## Discussion

We found that the surface waters around Australia are contaminated with small plastics that are mostly a by-product of the degradation of larger objects made of polyethylene and polypropylene. The high prevalence of plastic fragments smaller than 5 mm in Australian waters is consistent with other regions of the world's oceans, where microplastics were found to be the most abundant type of debris in all types of marine environment [8]–[10], [13], [41], [42]. Plastic pollution levels were moderate when compared to concentrations in other marine areas [8]–[10], [43], [44]. Higher amounts of plastic were found close to cities on Australia's east coast, as well as in remote locations (west Tasmania and North West Shelf). Recent studies reported toxicological effects of these small and contaminated plastics on a host of organisms, including large marine vertebrates [45] and fish [30]–[32], [46]. As such, small plastics are a type of harmful marine debris, implying that plastic hazards to Australian species and ecological communities are likely to be broader than those officially recognized.

### Characteristics of marine plastics

Captured plastic particles ranged in size from 0.4 – 82.6 mm. The frequency distribution of different sized plastics, which was skewed towards smaller particles, provides evidence for the existence of smaller plastics. Current methods for assessing plastic pollution at the ocean surface rely on the use of nets, which omits plastic particles outside the collectible range of their mesh [47]. It will be critical for future investigations to develop efficient and reproducible techniques capable of detecting smaller buoyant plastic particles (micro and nanoparticles). In addition, post processing techniques for sorting particles are also likely to miss small fragments [47]. An example of a new method with the potential to eliminate this limitation is the application of molecular mapping by reflectance micro-FT-IR spectroscopy, which does not rely on visual selection of plastic particles for characterization [48].

Hard plastics were by far the most common plastic type found (75.4%), but soft plastics (e.g. fragments of plastic wrappers) and lines (mostly fishing lines) were also relatively common (16.5% and 6.4%, respectively). It is interesting to note that soft plastics were more abundant in the larger size class (>2.4 mm). Our findings are consistent with recent studies documenting plastic pollution at the ocean surface, although explanations for variations in hard/soft plastic trends are not given [8], [10], [49]. Plastics gradually lose buoyancy in seawater as a result of biofilm formation [50]. We suggest that negative buoyancy due to biofouling occurs more quickly in soft/thin than in hard/thicker plastic fragments, resulting in a decline in the occurrence of soft plastics at the ocean surface, as they become smaller/older and begin to sink. Indirect evidence for this is that the proportion of soft plastics found in our coastal net stations was higher than that reported in open ocean settings further away from potential sources [49]. While a small number of experimental studies have confirmed that biofilms decrease the buoyancy of plastic items [50], [51], none of them report the magnitude or speed of this process across different types of small fragments.

The plastics reported here were mostly white/transparent (84.7%) or blue (8.3%), which is consistent with reports from other investigations on buoyant marine plastics [49], [52]. Depending on the feeding ecology of the affected animal, ingested plastic color proportions can differ from what is available in the environment [22]. For instance, ingested plastic color proportions in Australian shearwaters (*Ardenna pacifica* and *Ardenna tenuirostris*) are different from those reported by this study [20], [21]. As these birds are known to use color vision to select their food [21], [53], color can play a role in the ingestion risk associated with a certain plastic item. In contrast, the color proportion of plastics found in scats of fur seals (*Arctocephalus* spp.) at Macquarie Island (Australia) reflected what was available as flotsam in this environment [36]. These plastics are likely to be coming from the stomach contents of their main prey: the myctophid *Electrona subaspera*, which are pelagic small fish known to feed at night, selecting their food based on size rather than color [36].

The vast majority (98.5%) of the plastics detected were made of polyolefins (polyethylene and polypropylene), which is in agreement with what has been found for this size range of plastics in other marine regions around the world [47], [49]. Polyethylene and polypropylene account for most of our global plastic production (38% and 24%, respectively [6]) and they are typically applied in the manufacturing of single-use disposable packaging. In addition to packaging, which reaches the oceans primarily from coastal areas, fishing equipment made of these polyolefins (e.g. fish crates, nets, ropes, fishing lines [17]) are also likely sources of the plastic particles registered here. Other types of polymers found in this study include two pieces of expanded polystyrene (Styrofoam), a type of plastic also used in packaging and fishing gear, and one fragment of ethylene vinyl acetate, which has several applications such as the making of shoe soles and foam mats.

### Concentrations and sources

Our overall mean sea surface plastic concentration (*Cs*) was 4256.4 pieces km^−2^, which is similar to mean values reported for other regions outside subtropical gyres, such the Caribbean Sea (mean *Cs* = 1414 pieces km^−2^) and Gulf of Maine (mean *Cs* = 1534 pieces km^−2^) [9]. Within subtropical gyres, *Cs* values tend to be higher but within the same order of magnitude: 20328 pieces km^−2^ in the North Atlantic Gyre [9], and 26898 pieces km^−2^ in the South Pacific Gyre [8]. The exception seems to be the subtropical waters of the North Pacific and Mediterranean, which present mean *Cs* values that are an order of magnitude higher than those reported here: 116000 pieces km^−2^ in the Mediterranean [43], 174000 pieces km^−2^ in Northwest Pacific [44], and 334271 pieces km^−2^ in Northeast Pacific [10]. The latter is also known as the “Great Pacific Garbage Patch” [10], which is the largest aggregator of floating marine particles [54].

Our findings show that the distribution of marine plastics is quite widespread (93% of our net stations had at least one plastic piece), patchy (i.e. high variability within and between net stations' *Cs*) and dynamic (*Cs* ranged from 10% to 91% of *Ci*). Therefore, better spatio-temporal data coverage is required in order to identify plastic pollution hotspots within Australian waters. However, our data already indicate some spatial patterns: we observed high plastic concentrations close to Sydney and Brisbane cities. This suggests that plastics along Australia's east coast are mostly associated with domestic inputs. Since high quantities of plastic were also found close to Viti Levu (Fiji), we hypothesize that part of the plastics coming from coastal areas remain in the vicinity of their sources for a long time, while fragmenting into smaller pieces. This suggestion of local retention of plastic debris is in agreement with findings of recent studies (e.g.[7]) and could be tested by developing high-resolution models able to simulate plastic transport in coastal environments.

While the relatively high concentrations of plastic found close to the East Australian coast (net stations 18–20, 22, 37) seem to originate from local sources of plastics, those found in southwest Tasmania/eastern South Australia (net stations 5, 6, 8), and the North West Shelf (net station 54) could be associated with international sources and/or maritime operations. The presence of internationally-based plastics is suggested by (1) a fragment with Indonesian words that was collected in North West Shelf (see Figure 2) and (2) beach surveys, which registered in South Australia plastic debris from South Africa and South America [19]. High plastic concentrations in the southern tip of Tasmania (net station 5) might be caused by convergence effects of the encounter of the East Australian and Zeehan coastal currents [19], whereas those found off the east coast (e.g. net station 37) could be associated with meso-scale eddies of the East Australian current [55].

Aside from this study and the one that developed the biophysical model we applied here [15], we are not aware of any investigation that quantitatively considers the effect of vertical mixing processes on plastic concentrations. This effect needs to be taken into account in future studies assessing at-sea plastic pollution to allow better comparisons between data collected under different sea states. An important step towards improved simulations of plastic distribution along the water column is to better quantify the buoyant rise velocity (*w_b_*) of plastic particles from different oceanic and coastal surface waters. This variable has a considerable impact on the output of the model applied here. Furthermore, other environmental variables that were not taken into account in our one-dimensional column model (e.g. Langmuir circulation, breaking waves, mixed layer depth) could to be incorporated in this type of modeling.

### Potential pathways

The model outputs and routes taken by real drifters showed that plastics we found could have moved via a wide range of routes. This is because our net stations are within regions that experience different hydrodynamics (e.g. North West Shelf, Great Australian Bright, Coral Sea, Tasman Sea) [40]. Plastics have the potential to reach the sampled sites by travelling with a range of currents, including: (1) Antarctic Circumpolar current [56], which can carry plastics from a wide area to several of our net stations, particularly those along the coast of Tasmania, south coast of Australia, and Tasman Sea (net stations 1–15, 38 and 39); (2) South Equatorial current in the Pacific Ocean [56], [57], which can bring international plastics to the east coast of Australia (net stations 16–24, 40–45, 36, 37) and areas close to Fiji and New Caledonia (net stations 25–35); (3) East Australian current [55], [56], which can carry plastics from domestic highly populated regions (e.g. Brisbane and Sydney) to the net stations along the coast of Tasmania (net station 5, 15, 38, 39), east coast of Australia (net stations 16–24, 36, 37) and the Tasman Sea (net stations 1–4); (4) Holloway, Leeuwin, South Australian, and Zeehan coastal current systems [58]–[61], which can bring plastics from international areas connected to the Indonesian Throughflow and Indian Gyre (e.g. Southeast Asia/Indonesia [16]), as well as from domestic populated areas, to the net stations of the North West Shelf (net stations 48–57), off Perth (net station 14), and along the south coast of Australia, Bass Strait, Tasman Sea, and coast of Tasmania (net stations 1–13, 15–17, 37–39); and (5) West Australia current [61], which could transport international marine plastics that accumulated in the Indian Gyre to the net stations in the North West Shelf (net stations 48–57) and off Perth (net station 14).

It is important to note that running models backwards and using drifter trajectories arriving at sampled locations can only provide an indication of the directions that the collected plastics could have taken. To precisely estimate plastic pathways is quite challenging, mostly because plastic source locations and quantities are still largely unknown. Moreover, there are still no methods to estimate the “age” (drifting time) of a certain plastic particle. For instance, only plastics with long drifting times (years) could have matched the long tracks of drifters. Another limitation of the real drifter approach is that the resulting pathway formed by all drifter tracks arriving at a certain region is not only dependent on the ocean current systems, but also on the locations where most of the drifters were released. For instance, sampled sites in the North West Shelf (net stations 48–57) had only a few drifters arriving at them. This is mostly due to the non-existence of drifters in the shallow waters of the Indonesian archipelago. The creation of a shallow-water drifter (e.g. [62]) release program in this area could bring crucial information to help inform marine plastic pathways and sources.

### Final remarks

This investigation shows that the abundant and widespread small marine plastics around Australia are likely coming from a variety of domestic and international, land- and ocean-based sources. Even though marine plastic pollution is a global environmental issue, mostly caused by our massive production of plastic single-use disposable items, there are still no attempts to regulate plastic disposal on land at an international level [26]. Additionally, dumping of plastics into the oceans remains a significant issue owing the difficulties with regulation and enforcement [17], [63]. We suggest further at-sea studies on the characterization, spatial distribution, and pathways of marine plastics in coastal and oceanic regions around Australia, as well as on marine plastic toxin loads and interactions between small plastic particles and organisms at all trophic levels of the food web. This would improve our current knowledge on the effects of plastic on the marine ecosystem as a whole.

## Supporting Information

Table S1 **Net tow data (N = 171).** Columns indicate net station number, sampling date (day.month.year), location (degrees minutes), and sea surface plastic concentration (*Cs*; pieces per km^−2^).
(PDF)

Maps S1 **Cumulative probability distribution of virtual particles arriving at the 57 net stations.** The month when the virtual particles (25 per day) were released is indicated in each panel. Backtracking dispersal time was equal to 45 days and arriving destinations (net stations) are marked with red dots.
(PDF)

Maps S2 **Real drifter pathways arriving at the 57 net stations.** Purple dots indicate net station locations and asterisks indicate drifter release areas.
(PDF)
