# Supplementary material for: Marine Plastic Pollution in Waters around Australia: Characteristics, Concentrations, and Pathways
Source: PLoS One. 2013 Nov 27;8(11):e80466. doi: 10.1371/journal.pone.0080466 (PMC3842337; doi:10.1371/journal.pone.0080466)
Supplement: Table S1 — Net tow data (N = 171). Columns indicate net station number, sampling date (day.month.year), location (degrees minutes), and sea surface plastic concentration (Cs; pieces per km−2). (PDF) [file pone.0080466.s001.pdf]

Table S1

Net Station	Date (UTC)	Latitude (S)	Longitude (E)	Sea Surface Concentration (*Cs*)
1	10.06.11	39 18.663	163 35.799	653.51
1	10.06.11	39 18.663	163 35.799	691.96
1	10.06.11	39 18.663	163 35.799	566.79
2	11.06.11	39 57.089	160 25.080	950.45
2	11.06.11	39 57.089	160 25.080	2198.90
2	11.06.11	39 57.089	160 25.080	1217.12
3	12.06.11	40 38.087	155 53.425	1424.92
3	12.06.11	40 38.087	155 53.425	3254.64
3	12.06.11	40 38.087	155 53.425	0.00
4	13.06.11	41 08.068	152 17.007	785.90
4	13.06.11	41 08.068	152 17.007	0.00
4	13.06.11	41 08.068	152 17.007	1810.17
5	12.08.11	43 34.637	145 47.977	32167.51
5	12.08.11	43 34.637	145 47.977	29582.80
5	12.08.11	43 34.637	145 47.977	9081.73
6	13.08.11	41 55.318	144 32.515	24299.60
6	13.08.11	41 55.318	144 32.515	6077.41
6	13.08.11	41 55.318	144 32.515	0.00
7	14.08.11	39 02.484	142 25.125	3554.15
7	14.08.11	39 02.484	142 25.125	3211.54
7	14.08.11	39 02.484	142 25.125	2477.57
8	15.08.11	37 53.346	139 49.553	931.74
8	15.08.11	37 53.346	139 49.553	2513.76
8	15.08.11	37 53.346	139 49.553	13625.39
9	16.08.11	36 03.358	135 41.202	4177.68
9	16.08.11	36 03.358	135 41.202	2015.15
9	16.08.11	36 03.358	135 41.202	0.00
10	17.08.11	35 28.841	132 10.928	2332.49
10	17.08.11	35 28.841	132 10.928	0.00
10	17.08.11	35 28.841	132 10.928	1381.05
11	18.08.11	35 04.329	127 15.958	0.00
11	18.08.11	35 04.329	127 15.958	0.00
11	18.08.11	35 04.329	127 15.958	0.00
12	19.08.11	34 36.224	121 49.046	0.00
12	19.08.11	34 36.224	121 49.046	1603.96
12	19.08.11	34 36.224	121 49.046	1099.32
13	20.08.11	35 18.893	118 37.370	1540.86
13	20.08.11	35 18.893	118 37.370	0.00
13	20.08.11	35 18.893	118 37.370	2852.58
14	22.08.11	33 06.825	114 29.446	889.63
14	22.08.11	33 06.825	114 29.446	0.00
14	22.08.11	33 06.825	114 29.446	458.30
15	11.04.12	41 53.867	148 26.891	708.09
15	11.04.12	41 53.867	148 26.891	2732.41
15	11.04.12	41 53.867	148 26.891	2355.35
16	12.04.12	39 39.389	148 53.558	0.00
16	12.04.12	39 39.389	148 53.558	0.00
16	12.04.12	39 39.389	148 53.558	0.00
17	13.04.12	37 38.671	150 23.827	3823.59
17	13.04.12	37 38.671	150 23.827	3313.01
17	13.04.12	37 38.671	150 23.827	2840.54
18	13.04.12	35 24.230	150 50.390	8361.99
18	13.04.12	35 24.230	150 50.390	18324.00
18	13.04.12	35 24.230	150 50.390	6313.02
19	14.04.12	33 52.897	152 0.189	2903.60
19	14.04.12	33 52.897	152 0.189	7374.21
19	14.04.12	33 52.897	152 0.189	3858.96
20	15.04.12	33 10.310	152 41.536	5966.14
20	15.04.12	33 10.310	152 41.536	19119.87
20	15.04.12	33 10.310	152 41.536	33412.23
21	15.04.12	31 01.893	153 22.585	1954.41
21	15.04.12	31 01.893	153 22.585	3818.55

21	15.04.12	31 01.893	153 22.585	4404.43
22	16.04.12	28 20.522	153 55.265	9943.10
22	16.04.12	28 20.522	153 55.265	21021.85
22	16.04.12	28 20.522	153 55.265	32595.07
23	17.04.12	26 43.789	153 22.675	7064.40
23	17.04.12	26 43.789	153 22.675	4327.06
23	17.04.12	26 43.789	153 22.675	6168.01
24	04.05.12	24 55.084	155 12.525	743.14
24	04.05.12	24 55.084	155 12.525	0.00
24	04.05.12	24 55.084	155 12.525	0.00
25	06.05.12	23 52.232	162 20.311	5835.56
25	06.05.12	23 52.232	162 20.311	7695.14
25	06.05.12	23 52.232	162 20.311	10077.88
26	07.05.12	23 12.812	166 37.146	616.07
26	07.05.12	23 12.812	166 37.146	0.00
26	07.05.12	23 12.812	166 37.146	0.00
27	08.05.12	21 46.153	170 37.369	1248.23
27	08.05.12	21 46.153	170 37.369	8821.42
27	08.05.12	21 46.153	170 37.369	6114.52
28	07.06.12	18 33.897	176 30.406	48895.58
28	07.06.12	18 33.897	176 30.406	7857.04
28	07.06.12	18 33.897	176 30.406	3339.76
29	07.06.12	19 58.653	174 59.443	9647.43
29	07.06.12	19 58.653	174 59.443	7519.52
29	07.06.12	19 58.653	174 59.443	3789.07
30	08.06.12	21 16.990	173 34.862	946.73
30	08.06.12	21 16.990	173 34.862	801.95
30	08.06.12	21 16.990	173 34.862	1311.36
31	08.06.12	22 35.700	172 06.689	2575.43
31	08.06.12	22 35.700	172 06.689	2918.15
31	08.06.12	22 35.700	172 06.689	3507.91
32	09.06.12	23 44.992	170 32.263	0.00
32	09.06.12	23 44.992	170 32.263	0.00
32	09.06.12	23 44.992	170 32.263	0.00
33	09.06.12	25 04.307	168 42.713	2119.38
33	09.06.12	25 04.307	168 42.713	0.00
33	09.06.12	25 04.307	168 42.713	0.00
34	10.06.12	26 17.133	167 00.868	1423.41
34	10.06.12	26 17.133	167 00.868	764.70
34	10.06.12	26 17.133	167 00.868	729.39
35	10.06.12	27 31.614	165 11.757	711.58
35	10.06.12	27 31.614	165 11.757	2112.46
35	10.06.12	27 31.614	165 11.757	697.31
36	15.06.12	35 38.607	155 04.472	652.35
36	15.06.12	35 38.607	155 04.472	0.00
36	15.06.12	35 38.607	155 04.472	0.00
37	15.06.12	37 22.370	153 32.457	3594.90
37	15.06.12	37 22.370	153 32.457	9639.55
37	15.06.12	37 22.370	153 32.457	8349.88
38	16.06.12	42 16.869	148 58.254	5509.05
38	16.06.12	42 16.869	148 58.254	1845.78
38	16.06.12	42 16.869	148 58.254	0.00
39	17.06.12	42 49.848	148 26.515	1651.09
39	17.06.12	42 49.848	148 26.515	3050.52
39	17.06.12	42 49.848	148 26.515	2289.58
40	26.07.12	16 34.596	145 44.605	1960.78
40	26.07.12	16 34.596	145 44.605	5392.16
40	26.07.12	16 34.596	145 44.605	3921.57
41	27.07.12	15 01.649	145 23.440	2304.10
41	27.07.12	15 01.649	145 23.440	5172.33
41	27.07.12	15 01.649	145 23.440	13518.32
42	27.07.12	13 39.306	144 05.270	2398.91
42	27.07.12	13 39.306	144 05.270	956.41
42	27.07.12	13 39.306	144 05.270	1832.08
43	28.07.12	12 01.760	143 16.230	8954.09
43	28.07.12	12 01.760	143 16.230	3588.54
43	28.07.12	12 01.760	143 16.230	6582.30

44	28.07.12	10 44.893	142 20.410	3937.23
44	28.07.12	10 44.893	142 20.410	446.59
44	28.07.12	10 44.893	142 20.410	1382.16
45	29.07.12	12 15.928	141 37.748	2796.53
45	29.07.12	12 15.928	141 37.748	1559.97
45	29.07.12	12 15.928	141 37.748	450.97
46	30.07.12	15 29.180	141 02.689	0.00
46	30.07.12	15 29.180	141 02.689	596.58
46	30.07.12	15 29.180	141 02.689	0.00
47	30.07.12	17 02.883	140 46.650	0.00
47	30.07.12	17 02.883	140 46.650	0.00
47	30.07.12	17 02.883	140 46.650	0.00
48	17.08.12	18 39.076	119 29.745	2747.58
48	17.08.12	18 39.076	119 29.745	1114.00
48	17.08.12	18 39.076	119 29.745	0.00
49	17.08.12	18 51.743	118 40.746	2488.88
49	17.08.12	18 51.743	118 40.746	1110.76
49	17.08.12	18 51.743	118 40.746	534.60
50	17.08.12	18 58.975	118 12.687	4394.68
50	17.08.12	18 58.975	118 12.687	1703.95
50	17.08.12	18 58.975	118 12.687	1143.61
51	17.08.12	19 06.486	117 43.548	0.00
51	17.08.12	19 06.486	117 43.548	612.37
51	17.08.12	19 06.486	117 43.548	1854.75
52	17.08.12	19 25.826	115 55.023	3401.75
52	17.08.12	19 25.826	115 55.023	599.94
52	17.08.12	19 25.826	115 55.023	4049.19
53	19.08.12	20 23.058	116 23.058	7748.76
53	19.08.12	20 23.058	116 23.058	7266.80
53	19.08.12	20 23.058	116 23.058	39225.93
54	19.08.12	20 18.934	115 40.132	3249.67
54	19.08.12	20 18.934	115 40.132	1245.08
54	19.08.12	20 18.934	115 40.132	654.36
55	22.08.12	19 47.716	115 58.478	1241.92
55	22.08.12	19 47.716	115 58.478	2129.73
55	22.08.12	19 47.716	115 58.478	496.75
56	22.08.12	20 22.838	115 08.123	0.00
56	22.08.12	20 22.838	115 08.123	0.00
56	22.08.12	20 22.838	115 08.123	1294.44
57	25.08.12	21 50.655	113 54.765	12846.12
57	25.08.12	21 50.655	113 54.765	1932.06
57	25.08.12	21 50.655	113 54.765	3462.42
