# Supplementary figures and images for: Marine Plastic Pollution in Waters around Australia: Characteristics, Concentrations, and Pathways
Source: PLoS One. 2013 Nov 27;8(11):e80466. doi: 10.1371/journal.pone.0080466 (PMC3842337; doi:10.1371/journal.pone.0080466)

Maps S1

June 2011

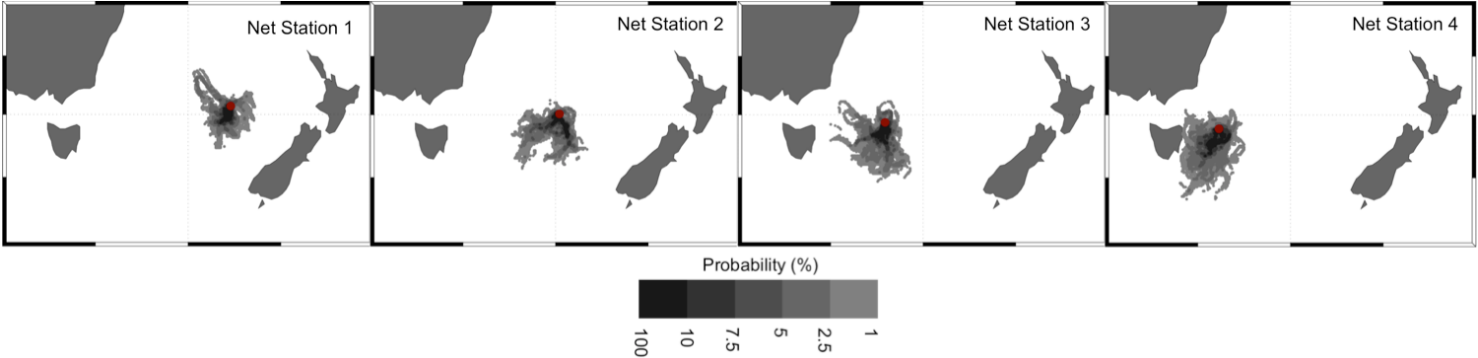

August 2011

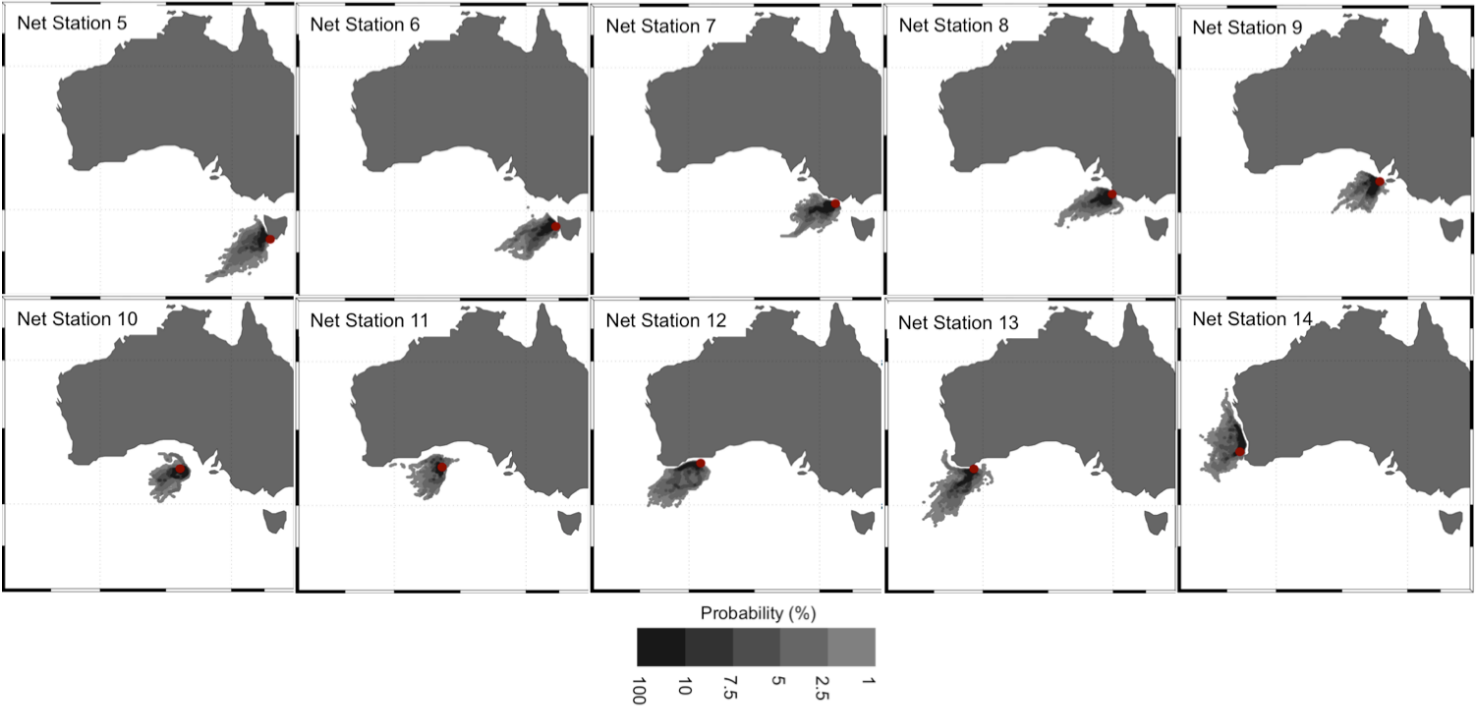

April 2012

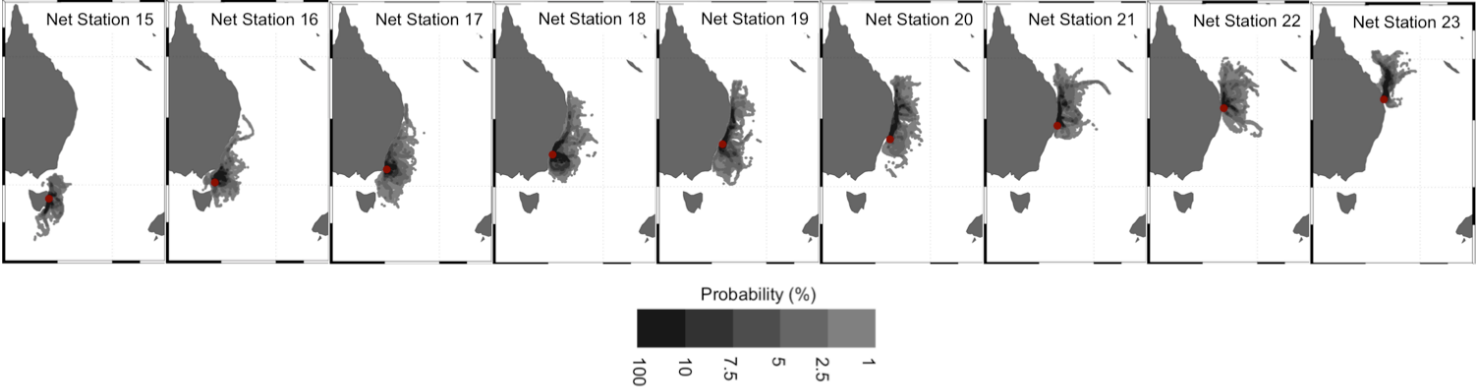

May 2012

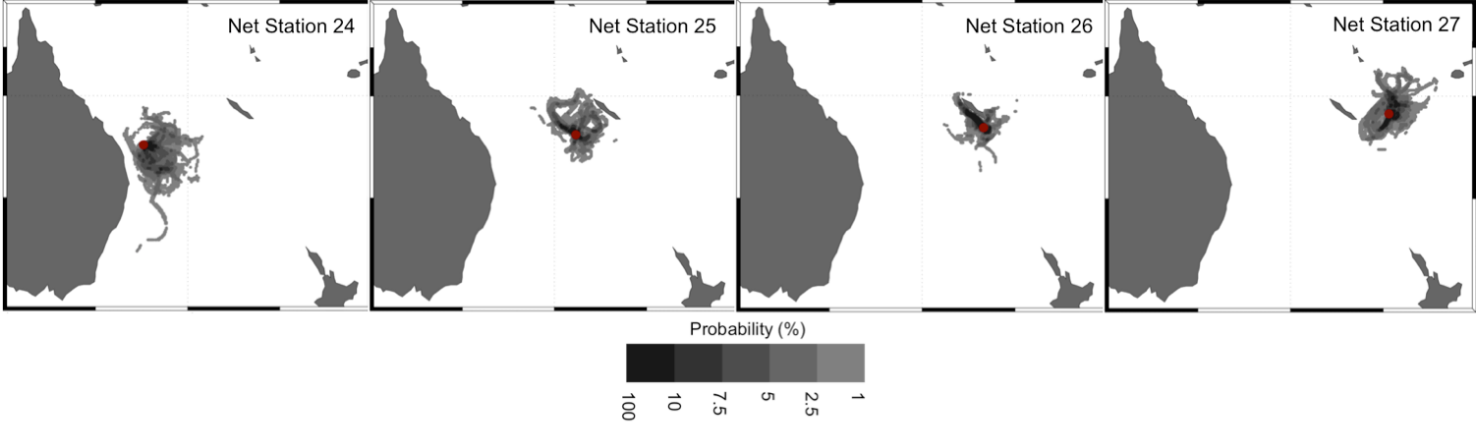

June 2012

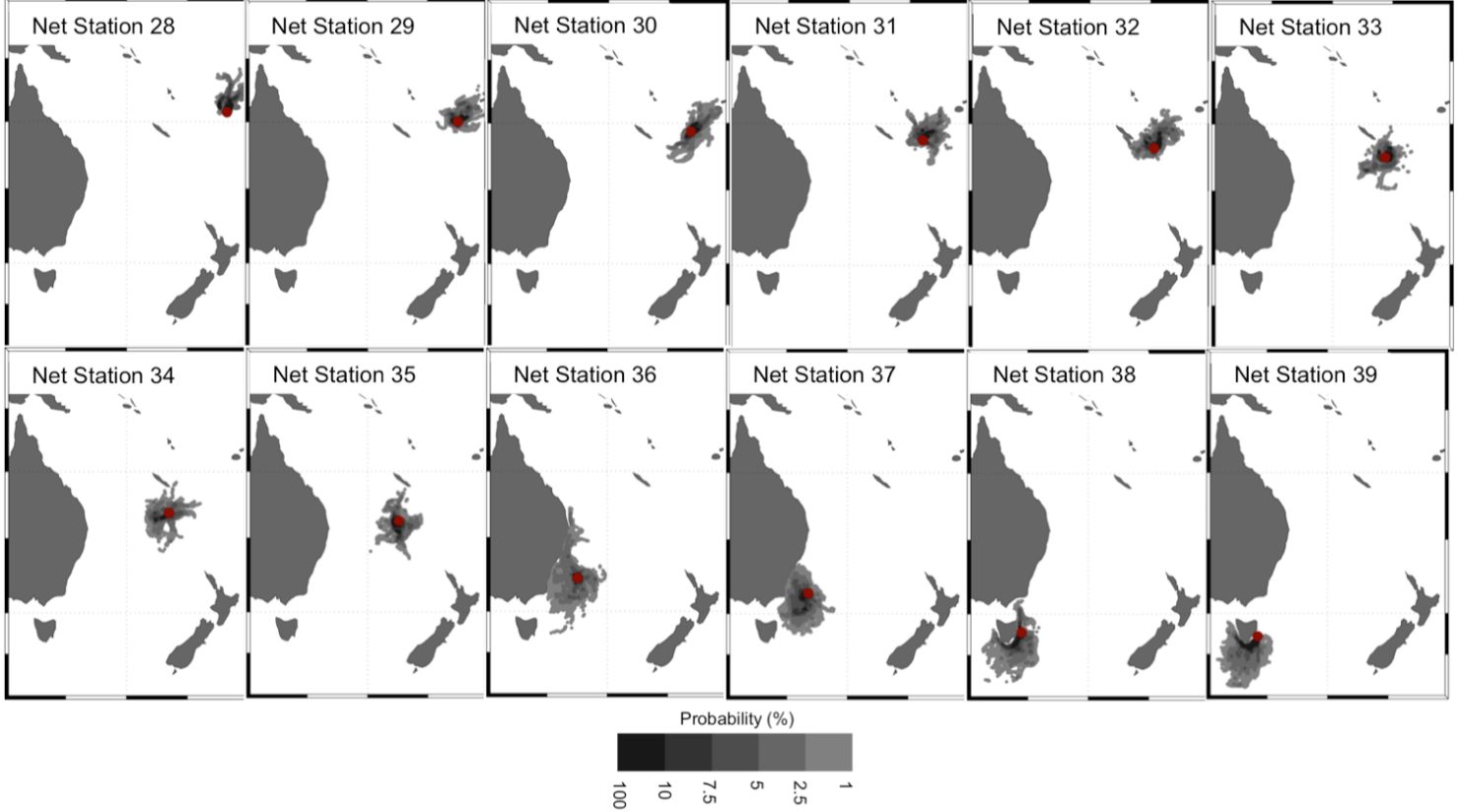

July 2012

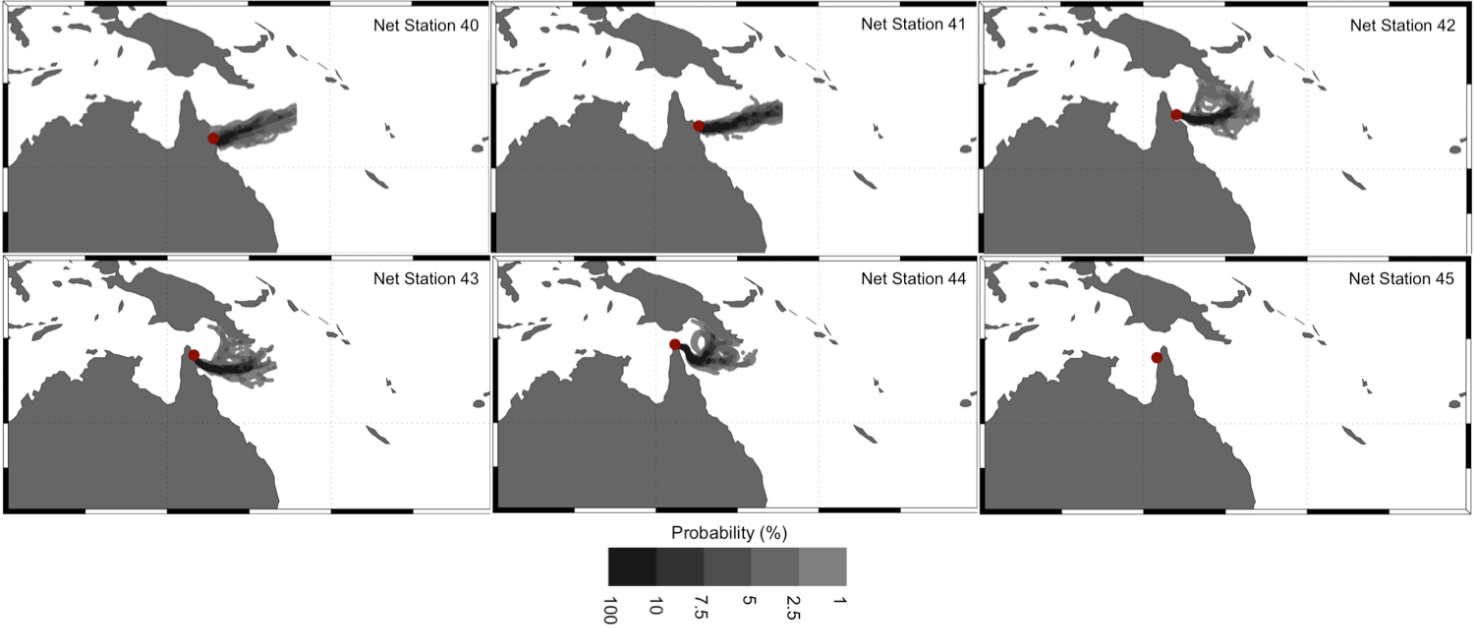

August 2012

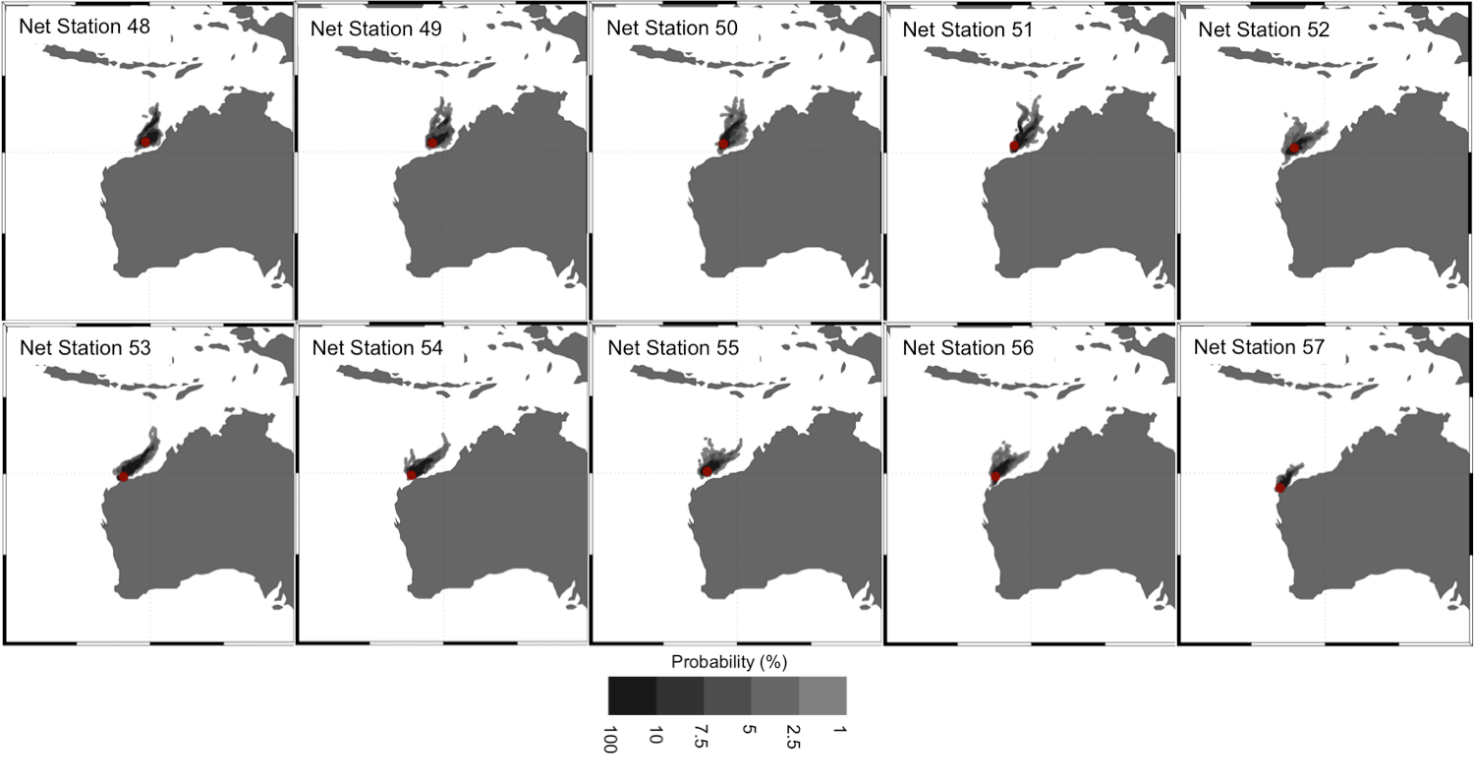

Supplement: Maps S1 — Cumulative probability distribution of virtual particles arriving at the 57 net stations. The month when the virtual particles (25 per day) were released is indicated in each panel. Backtracking dispersal time was equal to 45 days and arriving destinations (net stations) are marked with red dots. (PDF) [file pone.0080466.s002.pdf]

Maps S2

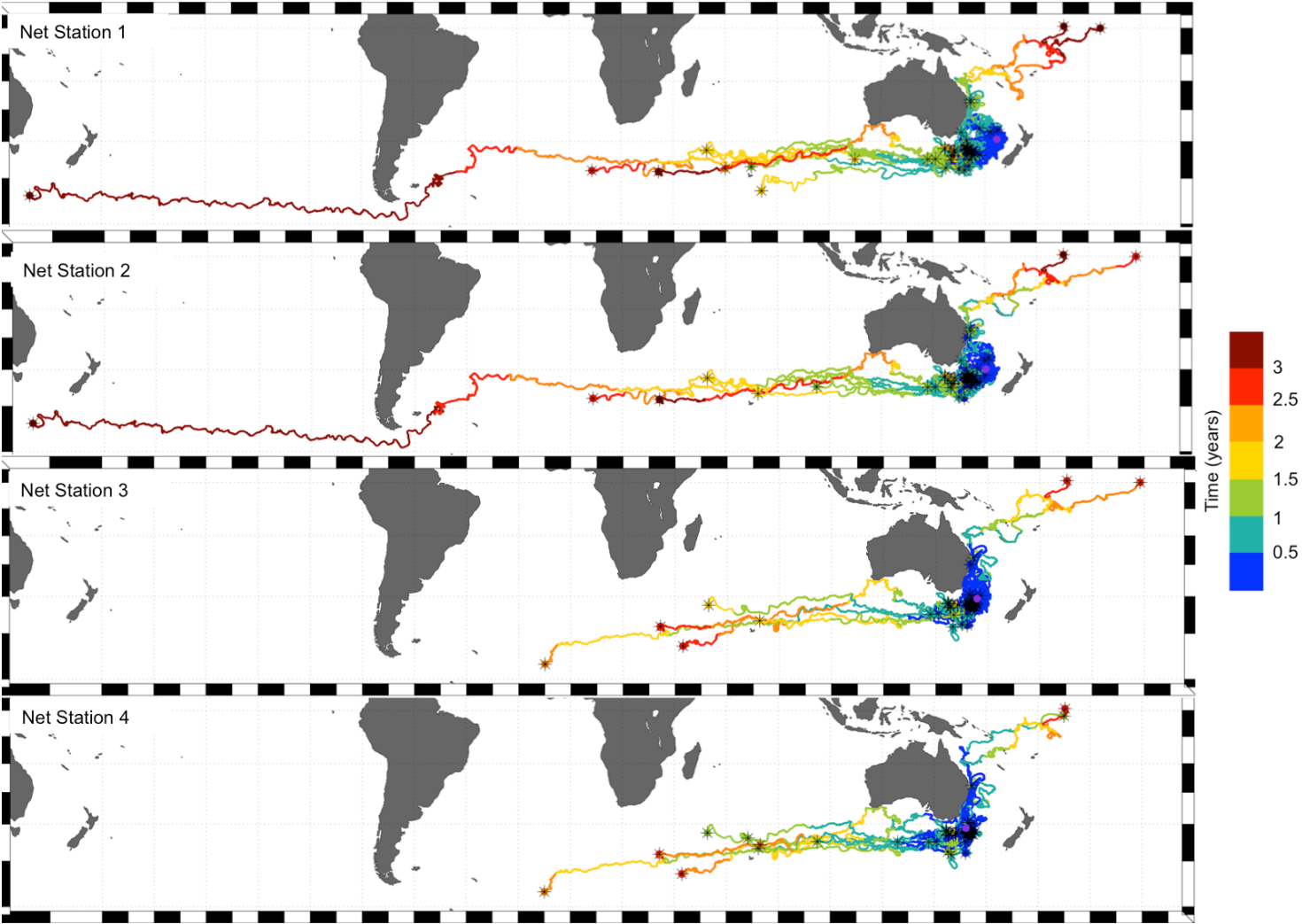

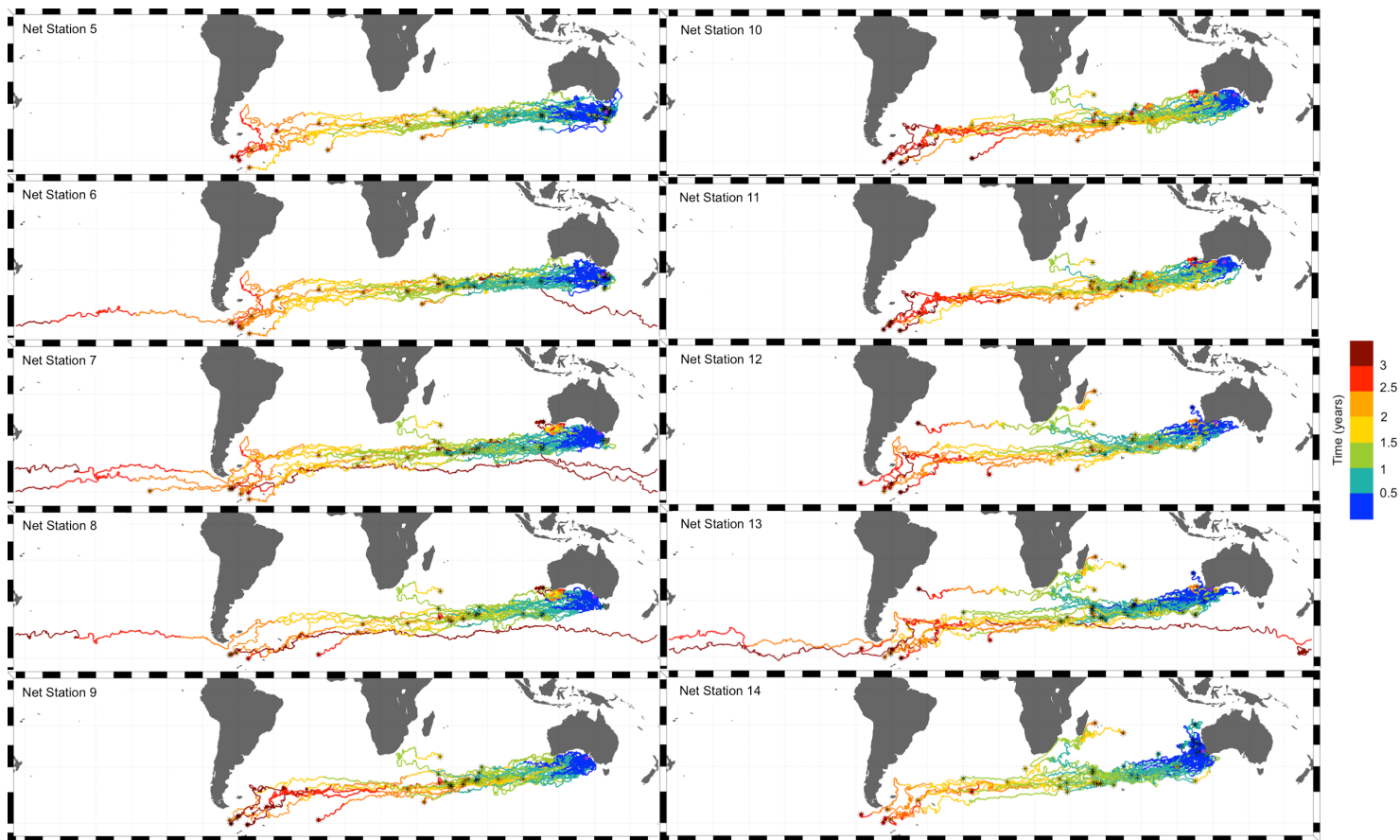

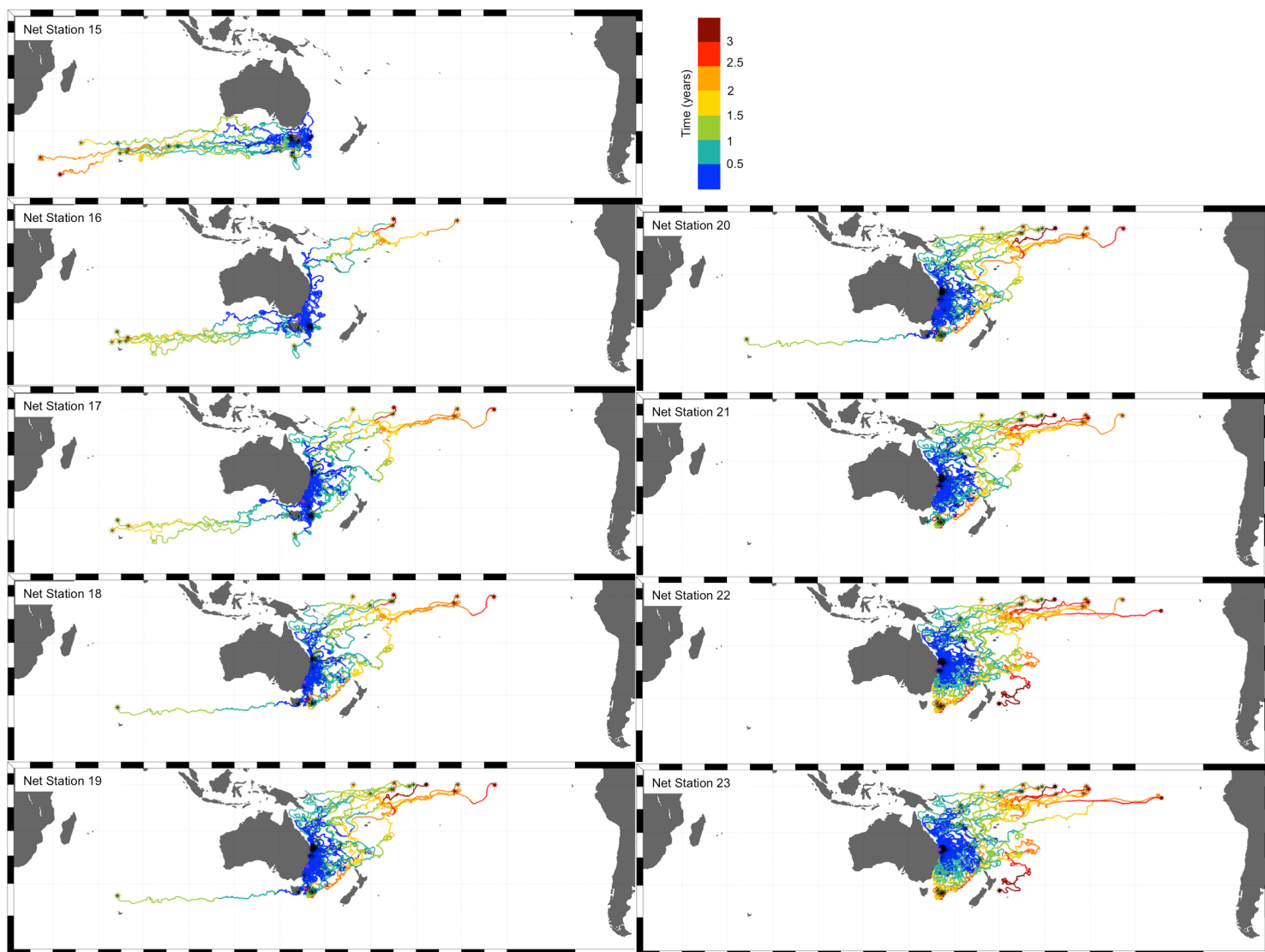

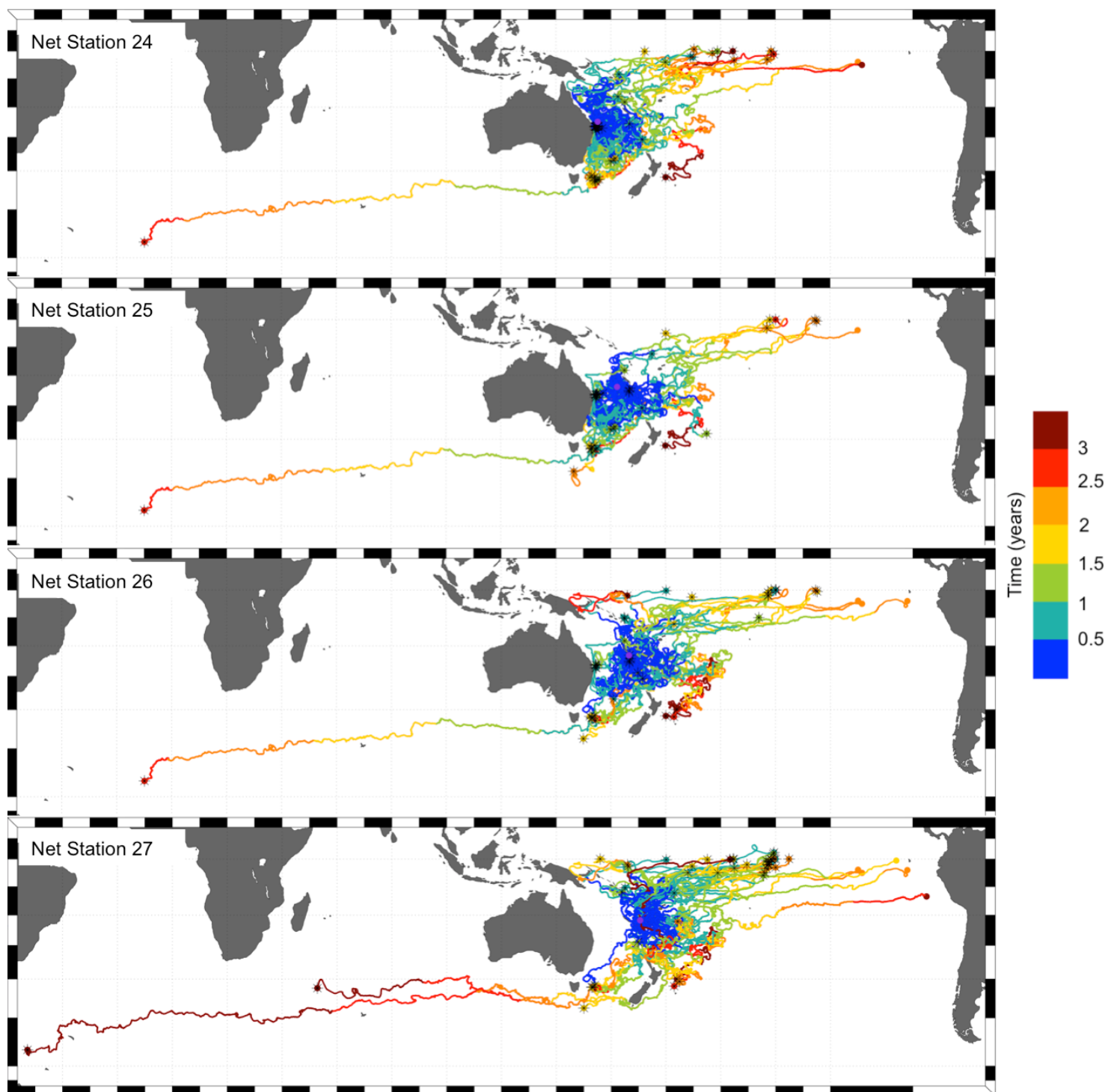

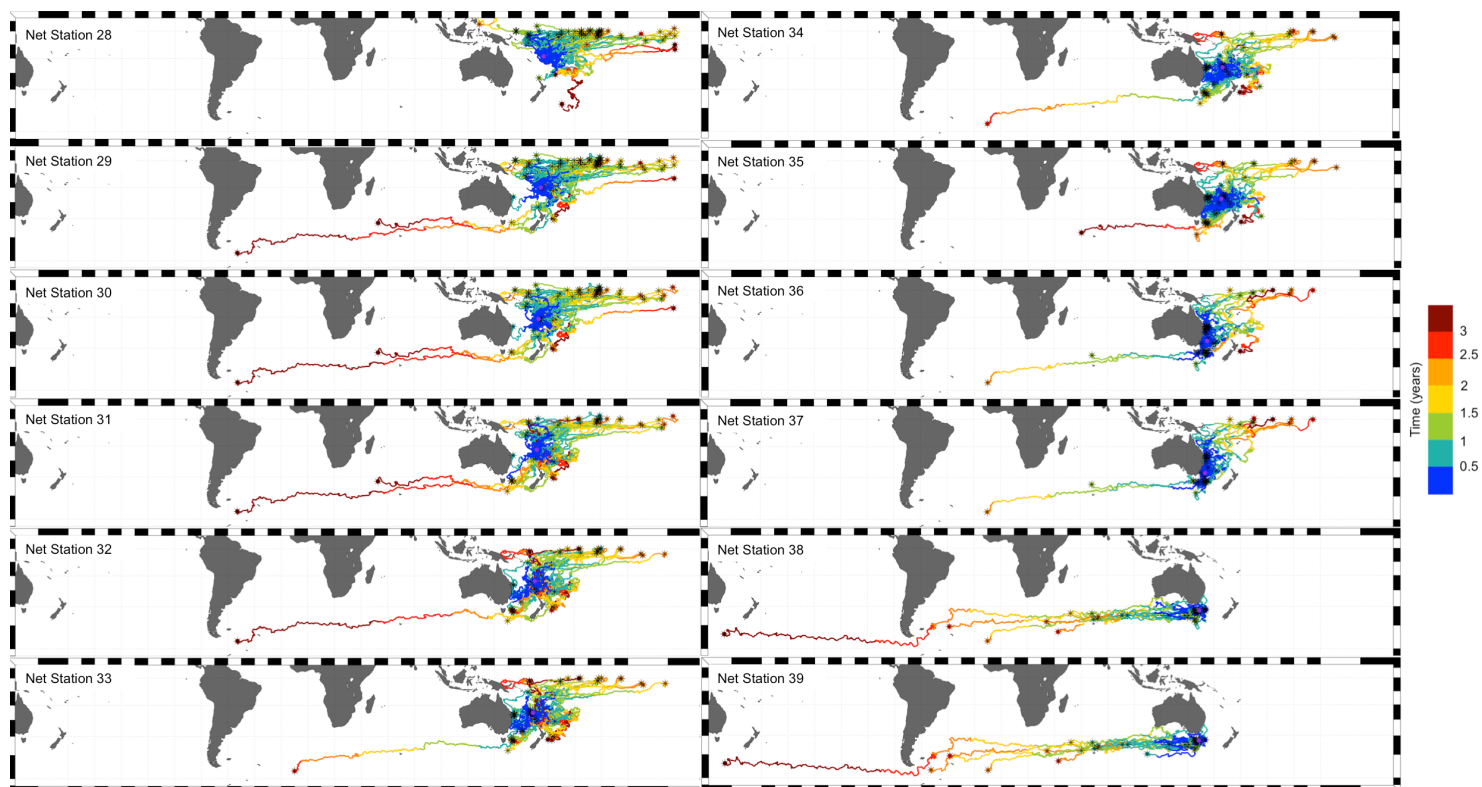

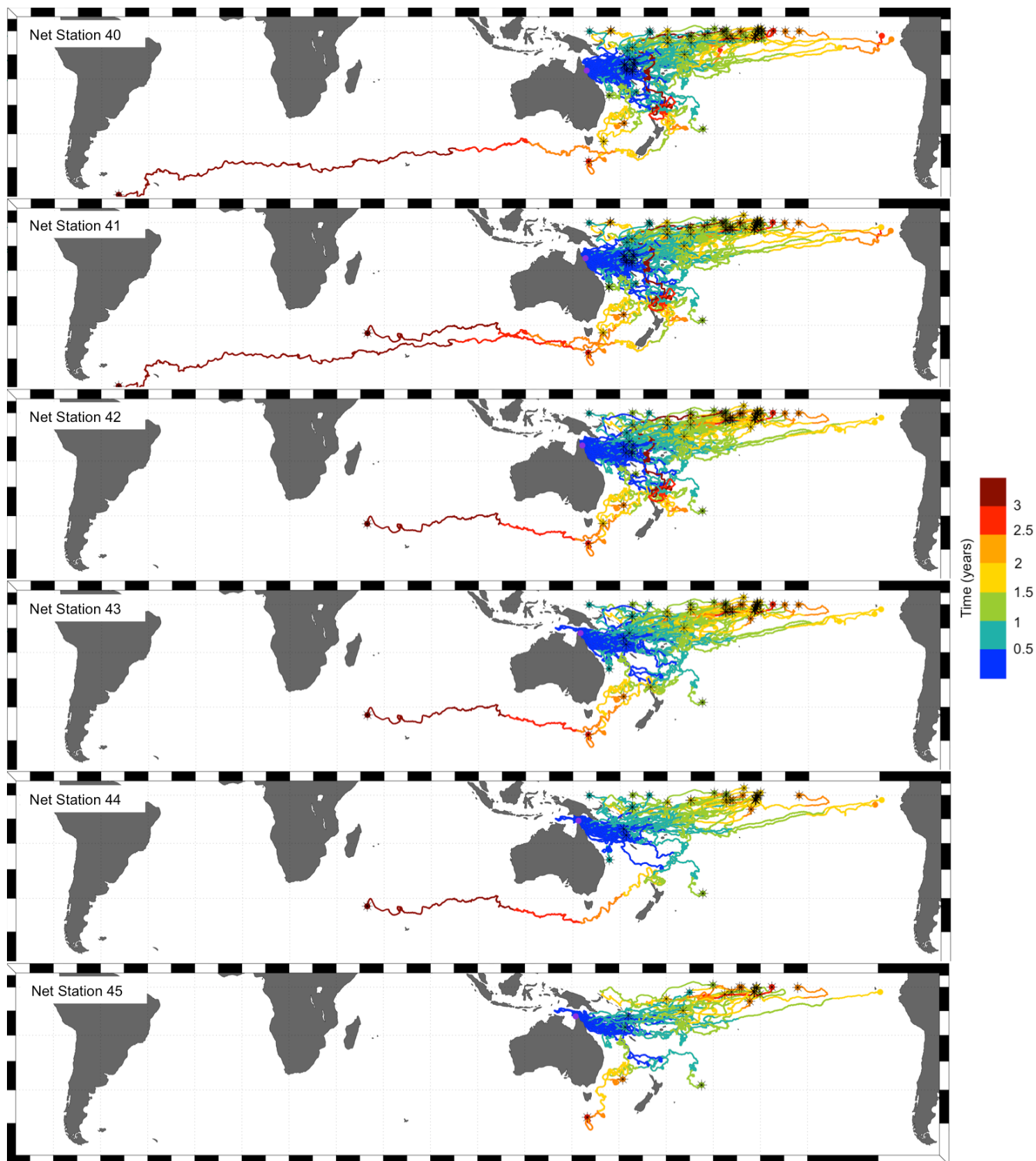

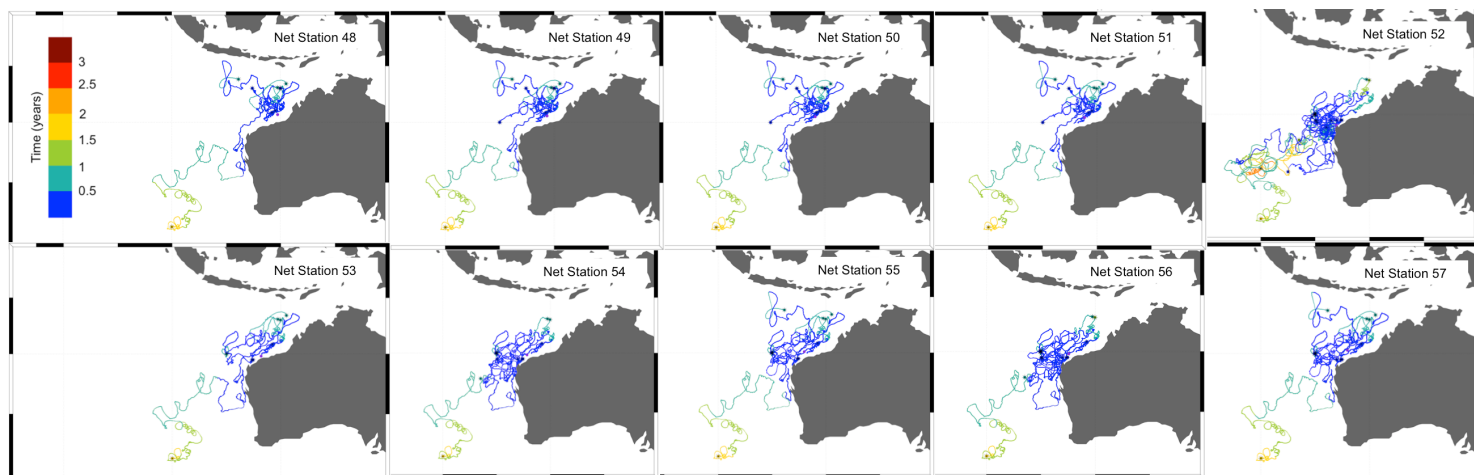

Supplement: Maps S2 — Real drifter pathways arriving at the 57 net stations. Purple dots indicate net station locations and asterisks indicate drifter release areas. (PDF) [file pone.0080466.s003.pdf]
